# Emergency Preparedness and Role Clarity among Rescue Workers during the Terror Attacks in Norway July 22, 2011

**DOI:** 10.1371/journal.pone.0156536

**Published:** 2016-06-09

**Authors:** May Janne Botha Pedersen, Astrid Gjerland, Bjørn Rishovd Rund, Øivind Ekeberg, Laila Skogstad

**Affiliations:** 1 Departments of General Surgery, Orthopedic Surgery, Anesthesia, Emergency Care, Intensive Care, and Obstetrics, Ringerike Hospital, Vestre Viken Hospital Trust, Drammen, Norway; 2 Departments of Anesthesia, Intensive Care, and Emergency, Baerum Hospital, Vestre Viken Hospital Trust, Drammen, Norway; 3 Department of Psychology, University of Oslo and Vestre Viken Hospital Trust, Drammen, Norway; 4 Department of Behavioral Sciences in Medicine, Institute of Basic Medical Sciences, Faculty of Medicine, University of Oslo, Oslo, Norway; 5 Department of Acute Medicine, Research and Development, Oslo University Hospital, Oslo, Norway; 6 Paramedic Sciences, Oslo and Akershus University College, Oslo, Norway; University of South Australia, AUSTRALIA

## Abstract

**Background:**

Few studies address preparedness and role clarity in rescue workers after a disaster. On July 22, 2011, Norway was struck by two terror attacks; 77 people were killed and many injured. Healthcare providers, police officers and firefighters worked under demanding conditions. The aims of this study were to examine the level of preparedness, exposure and role clarity. In addition, the relationship between demographic variables, preparedness and exposure and a) role clarity during the rescue operations and; b) achieved mastering for future disaster operations.

**Methods:**

In this cross-sectional study, healthcare providers (n = 859), police officers (n = 252) and firefighters (n = 102) returned a questionnaire approximately 10 months after the terror attacks.

**Results:**

The rescue personnel were trained and experienced, and the majority knew their professional role (healthcare providers *M* = 4.1 vs. police officers: *M* = 3.9 vs. firefighters: *M* = 4.2, *p* < .001, [scale 1–5]). The police officers reported significantly more lack of control (*p* < .001). In the multivariable analysis, being female (OR 1.4, *p* < .05), having more years of work experience (OR 2.3, *p* = < .001), previous training (OR 1.6, *p* < .05) and the experience of an event with > 5 fatalities (OR 1.6, *p* < .05) were all associated with role clarity, together with a feeling of control, not being obstructed in work and perceiving the rescue work as a success. Moreover, independent predictors of being more prepared for future operations were arousal during the operation (OR 2.0, *p* < .001) and perceiving the rescue work as a success (OR 1.5, *p* < .001).

**Conclusion:**

Most of the rescue workers were experienced and knew their professional role. Training and everyday-work-experience must be a focal point when preparing rescue workers for disaster.

## Introduction

Preparedness is a state of readiness to respond to crises [1], with one aim being to reduce negative outcomes. When preventive work is effective, it is invisible. Preparedness for disaster often includes a written emergency plan. The plan represents a structured definition of working roles and how to act in disaster situations. Such plans aim to clarify the working roles. Working during a disaster is a dynamic and ongoing process, and a written emergency plan, even though helpful, often does not cover the unfamiliar and unexpected demands that may occur in major disasters. During a disaster, it is important to obtain a balance between a standardization of behavior using plans as well as an ability to improvise and exercise professional judgment [2]. This is particularly critical when rescue workers are in situations of compromised security and uncertainty. Weisæth and Kjeserud [3] state that emergency planning and management of disasters cannot only be learned, but must be experienced in order to attain the knowledge one needs. They infer that very few people without training will be able to spontaneously manage a disaster or serious emergency, and conclude that readiness without training is of sparse value. Preparedness in rescue workers are obtained through an emergency plan, but even more through every-day experience with physically traumatized people either at the site of the accident or in hospital, through disaster drills or during simulation training with a team [4, 5].

Successful training will most likely have an effect on a better understanding of the tasks and roles during the operation, which may lead to less psychological distress during and after the operations, and even help mastering of future disaster operations. However, studies on the effects of training on rescue operations after terror attacks are limited. Some papers present an evaluation after a disaster drill, followed by suggestions for future events [6]. In a review studying the effects of disaster training, a lack of evidence between training interventions and improvement of knowledge and skills during a disaster response was found [7]. Few studies explore emergency preparedness per se and most of the work remains unreported in the literature. Some information and preliminary reports have been presented at conferences [8]. The predictors of post-traumatic stress symptoms in rescue workers after a disaster have been studied, but the results are conflicting regarding the relationship with prior experience, preparation and training as resilient factors [9].

On July 22, 2011, Norway was struck by two terror attacks. A car bomb was detonated in the Oslo Government district. Eight people were killed, many were wounded and governmental buildings were severely damaged. Approximately two hours after the first attack, the terrorist initiated shooting on a political youth camp on Utøya Island, where 69 were killed and many physically injured [10–12]. More than 500 people participated in the youth camp and were psychologically traumatized by the shooting [13]. The terror attacks of July 22, presented an unusual requirement for those who took part in the rescue operations and subsequent treatment of the wounded. Because so many were killed, wounded or emotionally traumatized, the pressure on rescue workers was demanding and extraordinary compared to their normal daily routines. In addition to facing severe human and material losses, the rescue workers also had to be prepared for the possibility of more terror attacks.

An ongoing disaster presents the possibility for real-life training. In this study we define rescue workers as personnel with professional education/training, affiliated either in the police force, fire department or in the emergency departments of a hospital. They are familiar with an emergency preparedness plan.

To the best of our knowledge, there is no systematic examination of preparedness of rescue workers and their response capabilities during large-scale disaster operations. On the basis of what is outlined above, we had two aims for the present study:

To examine

1. The level of preparedness, exposure and role clarity, and;
2. The relationship between demographic, preparedness and exposure and
  - Role clarity during the rescue operations and;
  - Achieved mastering for future disaster operations.

## Material and Methods

### Study design and setting

This cross-sectional study investigated professional personnel involved in rescue operations after the terror attacks in Norway. The present study is part of a larger project examining the challenges that the professional personnel met during these operations [14].

A questionnaire, which included background variables, contributions and exposure during the rescue operations, and how the events affected them, was sent to the rescue workers. The questionnaire was distributed approximately eight to 11 months after the terror attacks on July 22, 2011 (mean = 10 months) to personnel who worked with the terror victims or their relatives from July 22 to August 5, 2011, with a reminder sent one month after the first request. Those surveyed were kept anonymous. The questionnaire was distributed with an information letter while the return of the questionnaire was assumed to imply informed consent.

### Subjects

One could assume that personnel affiliated to an organization with a written plan were prepared for disaster, therefore three organizations with a consistent emergency preparedness plan were selected from the study population: healthcare professionals working in hospitals (n = 859), police officers (n = 252) and firefighters (n = 102). Police officers from three counties, firefighters from 10 independent units in four counties and healthcare providers from three hospitals were included. Among health care workers, 16% of the emergency medical service personnel worked close to the scene of terror. The corresponding numbers were 37% and 97% for police officers and the firefighters respectively. The other worked at their regular working place. The leader from each unit was responsible for the distribution and collection of the questionnaires. The respondent placed the completed questionnaire in an envelope. The envelope could be sealed, and were dropped in a sealed box. In the current paper, general and psychosocial healthcare personnel working within a hospital organization were merged into one group (n = 859). Personnel working in municipal emergency services (n = 72) and a center for next-of-kin outside a hospital (n = 95) were excluded, as they were unlikely to be working in an organization with a consistent emergency plan. Personnel working in a rehabilitation ward in one of the hospitals (n = 28) were excluded because their work started after August 5, and eight respondents with incomplete questionnaires were also excluded. The response rates were 54% among healthcare providers, 51% among police officers and 82% among firefighters.

### Assessments

Most of the items included in the questionnaire were developed by The Norwegian Centre for Violence and Stress Studies, and used in a cross-sectional study of Norwegian personnel mobilized during the 2004 Indian Ocean tsunami. A replica of some assessments from that study made it possible to compare data from two samples of Norwegian rescue workers [9].

#### Sociodemographic characteristics

Age, sex, duration of work at their current organization and working place during the rescue operation measured sociodemographic characteristics. These were classified as shown in Table 1.

**Table 1:** Background characteristics.

	Healthcare providers	Police officers	Fire-fighters	*P value*
Variables	n = 859	n = 252	n = 102	
**Gender**, male, n (%)	287 (33.6)	169 (67.9)	100 (99.0)	<.001**
< 30 years	144 (16.8)	29 (11.6)	11 (10.8)	
30–49 years	524 (61.3)	172 (69.1)	68 (66.7)	
>50 years	187 (21.9)	48 (19.3)	23 (22.5)	
< 1 year	44 (5.2)	19 (7.6)	2 (2.0)	
1–5 years	242 (28.4)	74 (29.6)	18 (17.6)	.015*
>5 years	567 (66.5)	156 (62.7)	82 (80.4)	
Work experience in similar tasks	541 (63.9)	163 (64.7)	70 (68.6)	ns
Training based on simulation	585 (68.8)	176 (69.8)	74 (72.5)	ns
Disaster drill	588 (69.2)	173 (68.9)	68 (66.7)	ns
Previous event with >5 fatalities	223 (26.1)	66 (26.3)	31 (30.4)	ns
Sites of terror	138 (16.2)	92 (36.8)	99 (97.1)	
Other (in hospital/out-patient emergency service. Centre for next of kin, patrolling, investigation)	713 (83.8)	157 (63.1)	3 (2.9)	<.001**

Note:

* p = <.05,

** p = <.001. Chi-square

#### Preparedness/training

Four items measured preparedness/training (see Table 1), and most items had three response alternatives: *1) yes—once*, *2) yes—several times*, *and 3) no*, *never*. The response alternatives were dichotomized into no and yes. The item, event with > 5 deceased, had two response alternatives, no and yes.

#### Perceived threat and inner psychological responses

Four self-designed items were used to assess possible perceived threats after the terror attacks. We questioned whether subjects experienced a (1) fear of explosion/shooting, (2) fear of being injured, or (3) other risks/uncertainty. The response alternatives for all items were: 0 = *no*, *not experienced*; 1 = *yes*, *but not stressful*; 2 = *yes*, *moderately stressful*; and 3 = *yes*, *very stressful* (Table 2). The items were summarized and named as perceived threats. Two questions assessed perceived psychological responses: (1) Did you feel overwhelmed, and (2) Did you feel a lack of control? These items were scored on a five-point Likert scale: 1 = *not at all* to 5 = *to a very high degree*.

**Table 2:** Exposure and psychological responses.

	Healthcare providers	Police officers	Fire-fighters	*p value*
Variables	n = 859	n = 252	n = 102	
**Witness experience** sum all items: yes	694 (80.3)	169 (67.1)	94 (92.2)	< .001**
Cronbach’s Alpha = 0.70				
**Perceived threats** (scale 0–3)	.5 (.5-.6)	.8 (.7-.9)	.9 (.7–1.1)	< .001**
Cronbach’s Alpha = 0.87				
**Feeling overwhelmed** (scale 1–5)	2.4 (2.4–2.5)	2.6 (2.5–2.8)	2.5 (2.3–2.7)	ns
**Feeling a lack of control** (scale 1–5)	2.1 (2.0–2.1)	2.5 (2.3–2.6)	2.1 (1.1–1.4)	< .001**
**Dissociation**	1.7 (1.7–1.8)	1.7 (1.6–1.8)	1.7 (1.6–1.8)	ns
Cronbach’s Alpha = 0.78				
**Arousal**	2.9 (2.9–3.0)	3.2 (3.0–3.3)	2.6 (2.4–2.8)	< .001**
Cronbach’s Alpha = 0.76				
(PCL) (scale 17–85) Median (range)	19.0 (17–64)	19.0 (17–69)	19.0 (17–64)	
PCL 35–50	18 (2.1)	7 (2.8)	1 (1.0)	
PCL > 50	3 (.4)	1 (.4)	2 (2.0)	
Cronbach’s Alpha = 0.91				

*Note*: Mean (95% CI) or n (%). Scale 1–5: 1 = not at all, 5 = to a very high extent for all items except threat and PCL,

** p = <.001. Chi-square or ANOVA

#### Exposure

Seven items measured exposure: witnessing disaster victims (1) searching for next- of-kin, (2) in despair at the campsite, (3) with major physical injuries, (4) seeing dead bodies, (5) having physical contact with dead bodies, (6) seeing torn body parts, and (7) strong smells or other sensory perceptions. All items were dichotomized into no and yes, with the sum of all items reflecting the number of positively scored items (0–7).

#### Dissociation and arousal

Eight items assessed dissociation and arousal during the rescue operations: a feeling of (1) “numbness”, (2) not being aware of the surroundings, (3) what you experienced was not real, (4) not being yourself, (5) not remembering what happened or only parts of it, (6) sharpened attention, (7) a reduced need for sleep and/or rest, and (8) positive activation (more energy or an intense sense of coping). A five-point Likert scale was used: 1 = *not at all* to 5 = *to a very high degree*. A factor analysis revealed two constructs: dissociation (items 1–5) and arousal (items 6–8).

#### PTSD symptoms

The PTSD Checklist (PCL-S) [15] was used to screen for posttraumatic stress symptoms (PTSD) and is a widely used and validated self-reported measure of PTSD [16]. Each item was scored on a five-point Likert scale: 1 = *not at all*, to 5 = *very often*, in which the overall scores ranged from 17 to 85. A cut off score of 31–38 has been reported to identify most cases with posttraumatic stress symptoms of clinical significance [17, 18] and a cut-off score of 50 on the PCL scale has been recommended for possible PTSD (15). That is the reason for the selection, but lower scores have also identified persons with possible PTSD (17).

#### Work-related variables

The items: “Working with adequate resources to perform the rescue work satisfactorily” and “Know your professional role/working tasks” (Role clarity) were measured on a five-point Likert scale: 1 = *not at all* to 5 = *to a very high degree* [median split score: < 4 *(to a low degree)* and ≥ 4 *(to a high degree)*]. The latter item was used as an outcome in the regression analysis. A Likert scale was used on the items, “Comprehend people’s reactions better” and “More prepared to master similar situations after the rescue work”. The latter was dichotomized and used as an outcome in the regression analysis [median split score: <3 (*to a low degree)* and ≥ 3 (*to a high degree*)].

#### Evaluation of the rescue work

Seven items were used to evaluate the rescue work: To what degree did you find the rescue work a) successful, b) meaningful, c) I/we fell short, d) I/we were hindered, e) competition was a problem, f) I received sufficient advice and support, and g) I stretched myself too far because a high effort was expected. The items were measured on a five-point Likert scale: 1 = *not at all* to 5 = *to a very high degree*, with a factor analysis identifying two factors: successful (items a, b, f) and obstructed (c, d, e, g).

### Ethics

Prior to the study, the Data Protection Officer at Oslo University Hospital was contacted to clarify further approval from the regional Ethics Committee. The Data Protection Officer informed that further approval from The Regional Ethics Committee was not necessary due to anonymously collected data. The data was stored on the research server at the hospital.

### Data analyses

The data was presented as means with 95% confidence intervals, which means the alpha was set at 5% (0.05), or percentages, and in general there were few missing data (1–2%). Where appropriate, the variables were dichotomized. Chi-squared and Kruskal–Wallis tests were used to compare proportions; whereas independent samples *t* tests were used to compare means. A logistic regression analysis (Forward Wald) identified the predictors of role clarity and being more prepared to master future disaster operations, and SPSS statistical software (version 21; SPSS, Chicago, IL) was used.

## Results

### Previous training and work experience

Approximately two-thirds of all respondents reported previous training in either work experience from similar tasks, simulations or disaster drills (Table 1). In addition, more than one-fourth reported to have participated in a rescue event involving more than five fatalities, and there were no significant differences between the groups in any of these items. A total of 99 (97%) of the firefighters worked at the sites of terror, while approximately half of the police officers had other duties such as office work, patrolling and investigation. Most of the healthcare providers 713 (84%) worked in a hospital.

### Perceived threat and psychological responses

The level of perceived threat (fear of shooting, fear of getting hurt or other danger) was generally low (Table 2). Healthcare providers perceived less of a threat during the rescue work compared to police officers and firefighters (*p* < .001) and the police officers reported significantly more lack of control (*p* < .001). More firefighters and healthcare providers witnessed victims compared to the police officers (p < .001). Police officers perceived the highest level of arousal, and healthcare providers perceived more arousal than firefighters (*p* < .001). Few responders in either group had post-traumatic stress symptoms at clinical sub-threshold (1.0–2.8%) or at a PTSD level (0.4–2.0%) (ns).

### Work-related variables

Table 3 shows work-related variables. The great majority knew their professional role (role clarity) (healthcare providers *M* = 4.1 vs. police officers: *M* = 3.9 vs. firefighters: *M* = 4.2, *p* < .001). The police officers reported less adequate resources (*M* = 2.8 vs. firefighters: *M* = 3.8 and healthcare providers *M* = 4.1; *p* < .001), as well as more often perceiving the rescue work to be obstructed as in falling short, being hindered, experiencing competition or stretching themselves too far (*M* = 2.4 vs. firefighters: *M* = 1.9 vs. healthcare providers *M* = 1.9, *p* < .001). All groups reported the rescue work to be successful, but the police officers significantly less so (*M* = 3.7 vs. firefighters: *M* = 4.2 vs. healthcare providers: *M* = 4.0, *p* < .001). All groups reported being more prepared to master similar situations in the future (*M* = 3.5–3.6), and with no significant differences between groups.

**Table 3:** Working role, resources and evaluation of the rescue work.

	Healthcare providers	Police officers	Fire-fighters	*p value*
Variables	n = 865	n = 253	n = 102	
**Role clarity**	4.1 (4.0–4.1)	3.9 (3.8–4.0)	4.2 (4.0–4.3)	< .001**
**Working with adequate resources**	4.1 (4.0–4.2)	2.8 (2.6–2.9)	3.8 (3.6–3.9)	< .001**
**The rescue work was obstructed** (Scale 1–5): (fell short, hindered, competition, stretch myself too far)	1.9 (1.8–1.9)	2.4 (2.3–2.5)	1.9 (1.8–2.0)	< .001**
**The rescue work was successful** (Scale 1–5): (Successful, meaningful, sufficient advice and support)	4.0 (4.0–4.1)	3.7 (3.6–3.7)	4.2 (4.1–4.3)	< .001**
**More prepared to master similar situations in the future**	3.6 (3.6–3.7)	3.6 (3.5–3.7)	3.5 (3.3–3.6)	ns

*Note*: Mean (95% CI) or n (%). Scale: 1 = not at all, 5 = to a very high extent.

** p = <.001. ANOVA

### Predictors of role clarity

Eighteen variables were univariately significantly associated with role clarity during the rescue work (Table 4). In the multivariable analysis, being female (OR 1.4), more years of work experience in the current organization (OR 2.3–3.5), experiencing training based on simulation (OR 1.6), having previously experienced an event with > 5 fatalities (OR 1.6), not feeling a lack of control (OR .8), not being obstructed (OR .4) and perceiving the rescue work as a success (OR 2.3) were all associated with role clarity. There were no significant differences across professional groups.

**Table 4:** Predictors of role clarity during the rescue work.

Variables	Univariable (unadjusted)			Multivariable (adjusted)		
	OR	95% CI	*p-value*	OR	95% CI	*p-value*
**Gender** (female vs. men)	1.3	1.0–1.8	.036*	1.4	1.0–2.0	.041*
30–49 years	(reference group)1.5	1.0–2.2	.026*			
>50 years	1.7	1.1–2.6	.024*			
< 1 year	(reference group)					
1–5 years	2.0	1.2–3.5	.011*	2.3	1.3–4.3	.006*
> 5 years	3.6	2.1–6.0	<.001**	3.5	1.9–6.2	<.001**
**Rescue worker group**: Police officers	(reference group)					
Healthcare providers	1.8	1.3–2.5	<.001**			
Fire-fighters	2.6	1.4–4.7	.002*			
Previous work experience	1.4	1.1–1.9	.015*			
Training based on simulation	1.6	1.2–2.1	.001*	1.6	1.1–2.2	.007*
Previous disaster drill	1.5	1.1–2.0	.004*			
Previous event with > 5 fatalities	1.7	1.2–2.4	.002*	1.6	1.1–2.5	.019*
Working with adequate resources (high vs. low)	3.1	2.3–4.1	<.001**			
Perceived threat	.8	.7–.9	.004*			
Witnessing			ns			
Present at the site of terror (yes vs. no)			ns			
Arousal			ns			
Dissociation	.7	.5–.9	.003*			
Felt overwhelmed (scale 1–5)	.7	.6–.8	< .001**			
Felt a lack of control (scale 1–5)	.6	.5–.7	< .001**	.8	.7–.9	.003*
**Satisfaction**						
Perceived success in the rescue work (scale 1–5)	2.9	2.3–3.7	< .001**	2.3	1.7–3.0	<.001**
Perceived obstruction in the rescue work (scale 1–5)	.3	.3–.4	< .001**	.4	.3–.5	<.001**

*Note*: Logistic regression analysis, forwald wald.

* p > .05,

** p > .001.

The dependent variable is dicotomized: 0 = 1–3: 1 = 4–5 on the 1–5 scale

### Predictors of being more prepared to master future operations

Four variables were univariately significantly associated with being more prepared to master similar situations in the future. In the multivariable analysis, being aroused (OR 2.0) and perceiving the rescue work as successful (OR 1.5) were both independent predictors.

## Discussion

Most respondents were experienced, had been working in their current organization for at least five years and had participated in disaster training and/or drills. The prevalence of possible PTSD was very low. A high level of role clarity was significantly associated with work-related variables such as time working at their current organization, disaster training and/or prior experience from mass casualty events with more than five fatalities. Role clarity was also associated with a feeling of control, that the rescue work was perceived as a success, and with female respondents. When perceived mastering for future disasters was examined, there was an association between higher levels of arousal and the rescue work being perceived as a success. Moreover, there were no statistically significant differences between professional groups when predictors were taken into account.

### Role clarity

Two key areas were associated with a higher level of role clarity: 1) preparedness, and 2) the perception of control, and rescue work success.

#### 1) Preparedness

We wanted to study personnel working in organizations with an emergency preparedness plan. A written plan is not sufficient to handle a disaster, and the local organizations are responsible for training and drills to prepare for emergencies. Each department within an organization must implement the plan according to its specific functions and needs. Every large-scale event is different [19] and improvisation is necessary in order to meet specific demands.

Being prepared strengthens the ability one has to cope during a chaotic situation ^8^, and everyday training and hands-on practice give an opportunity to develop skills and to be more confident with a given role. Based on preparation and training, when a disaster strikes, the personnel can perform everyday work with an adaptation to the particular large-scale event at hand [20]. In the present study, the respondents were experienced, and most had been trained in emergency preparedness. These factors were associated with higher role clarity, which may support Weisæth and Kjeserud [3] who emphasize that readiness without training is of sparse value. In addition, the terror sites were situated in areas with large populations, hence increasing the odds of experienced personnel. In Scandinavia, a number of hospitals receiving critically injured patients are offered systematic and regular trauma training [5]. Multidiciplinary systematic large-scale disaster drills are part of the training for rescue personnel [21]. This may reflect the significant relationship between preparedness and a higher degree of role clarity during the terror events, and thereby highlight the importance of regular systematic training. This is in accordance with studies of systematic team-based training in trauma care that showed beneficial in-hospital effects [4], well-functioning cooperation [22] and increasing role clarity after systematic multidisciplinary team training [23].

On the other hand, a systematic review found insufficient evidence for the effectiveness of training interventions to help improve knowledge and skills during an exercise [7]. That review included five papers, which studied out-of-hospital healthcare providers. The studies were from heterogenic samples working in different organizations, which may explain the different outcome relative to our study.

#### 2) Perception of control and success

A greater sense of control was also associated with a higher level of role clarity. It is therefore likely to assume that there is a connection between a sense of control in performing everyday routines (preparedness) and being confident in performing similar tasks during a disaster. The police officers reported less control than the other groups. Some of those who worked at the sites of terror faced extremely difficult tasks, such as risk of collapse of buildings, risk of other terror attacks, an overloaded rescue boat, long response time for the police helicopter and guidelines to hold the other rescuers back because of the unsecured situation with the terrorist A feeling of success also had a significant association with role clarity, which can be attributed to practiced routines, and a corresponding greater sense of control. Helping victims during the senseless terror attacks was a positive experience for many professionals.

Female gender was also associated with role clarity when we analyzed the groups together. Almost all firefighters were men, so we performed a regression analysis for each group separately. Female gender was not a significant predictor in any of the groups, but became significant when all groups were analyzed together. We do not have any data to explain this, but more women worked as healthcare providers relative to the other groups. Despite a demanding situation and unusual tasks, role clarity may be related to working in familiar surroundings within the hospital with well-known equipment and sufficient recourses.

### Better prepared to master similar situations in the future

Arousal and a feeling of success were associated with a sense of being better prepared to master similar situations in the future. The degree of arousal was moderate and may have contributed to feelings of mastery, control, and thus to a sense of a successful operation. Under demanding conditions, a low level of arousal can be a sign that the work was perceived as being routine. In this special situation, there were many unknown events, but the rescue personnel had a high level of knowledge and training, which may have influenced their level of arousal. One can wonder if a high level of arousal can contribute to a feeling of loss of control, and hence a negative experience that could subsequently reduce the learning effect. A positive experience can confirm that one has handled a situation properly, something that will increase confidence in managing similar situations. One study supports this conclusion [24], finding that disaster training increases one’s ability to cope with complex situations.

### Post-traumatic stress symptoms

Personnel in all groups were exposed to highly traumatic experiences, though the prevalence of possible PTSD (PCL-S >50) was low (0.6%), and with no significant differences between the groups. This is even lower than the findings in policemen after the 2004 Madrid train bombings (3.9%) [25]. A large number of the rescue personnel in the present study witnessed people in despair and injured or deceased victims, and even felt threatened, but few reported this as very/extremely stressful. This may be the result of the extensive training and experience among the rescue workers [26]. Among deployed personnel after the 2004 Indian Ocean tsunami [9] preparedness, including training in similar tasks and exposure to mass casualty events, was also related to a lower degree of PTSD. Most of the rescue personnel were offered debriefing and/or peer-support after the duty. This may have partly influenced the low rates of possible PTSD. We find it however, more likely that the main reason for the low rate of possible PTSD was that the staff was well prepared and had high role clarity. It is unlikely that a single debriefing should be sufficient to prevent PTSD.

### Strengths and Limitations

The moderate to high response rate was considered to be an advantage of the present study, especially for the firefighters, while the use of a well-known questionnaire such as the PCL-S was also a strength. Lower response rate among healthcare providers and police may represent a selection bias. We have little information on non-responding personnel. The variables “preparedness” and “role clarity” had only one item, which may be considered a limitation, even though we think it should be clear to the participants what is meant by the question. More detailed questions about working tasks, responsibilities and work exposure could have yielded additional information (e.g. interview), but this would have required considerable resources that were not available. However, it is unlikely that this would have changed our main finding, namely that the personnel were experienced and trained, and that preparedness was associated with role clarity.

Respondents on sick leave might not have received the questionnaires, which may have biased the results. We did not perform assessments of previous or current mental problems, coping styles or personality traits. In addition, we did not determine the marital status or educational levels of the participants, or whether they had lost someone close during the attacks. Even so, it is unlikely that this would have altered the main findings of the study.

The participants completed the questionnaire 8–11 months after the event. Time elapsed from the event to inclusion may represent a recall bias. It may be considered a limitation of this study that we did not conduct a prospective study with at least two time points. The development of e.g posttraumatic stress symptoms could then been found. On the other hand, a prospective design could not be anonymous, which would probably have lowered the response rate. By determining the symptoms after almost one year, we obtained data that demonstrated the long-term effects of the event.

### Clinical Implications

The majority of the rescue workers had been trained and prepared to handle both everyday and large-scale mass casualty events. There was an association between this and role clarity, but also between role clarity and perceiving control and success. The low level of post-traumatic stress symptoms indicates that the rescue workers who participated were quite resilient, and there are reasons to believe that this also may be associated with preparedness. This strengthens the importance of preparedness, not only in relation to the importance of emergency preparedness plans, but especially for training within the organization.

## Conclusion

The rescue workers who participated in the terror operations in 2011 were exposed to high levels of death, injuries and destruction. Most personnel within all groups were experienced and prepared for handling traumatic events. Preparedness predicted a higher perceived role clarity together with a perception of feeling in control. Perceiving the work as successful predicted both a higher role clarity and preparedness for future incidents. Preparedness provides role clarity, and must be prioritized in training programs among rescue workers.
